# Investigation of resonance-stabilized radicals associated with soot particle inception using advanced electron paramagnetic resonance techniques

**DOI:** 10.1038/s42004-023-00896-4

**Published:** 2023-05-24

**Authors:** Jessy Elias, Alessandro Faccinetto, Hervé Vezin, Xavier Mercier

**Affiliations:** 1 Université Lille, CNRS, UMR 8522-PC2A-Physicochimie des Processus de Combustion et de l’Atmosphère, F-59000 Lille, France; 2 French Environment and Energy Management Agency, Angers, France; 3 Université Lille, CNRS, UMR 8516-LASIRe-Laboratoire de Spectroscopie pour les Interactions, la Réactivité et l’Environnement, F-59000 Lille, France

**Keywords:** Chemical physics, Analytical chemistry, Environmental chemistry, Pollution remediation

## Abstract

In order to tackle the climate emergency, it is imperative to advance cleaner technologies to reduce pollutant emission as soot particles. However, there is still a lack of complete understanding of the mechanisms responsible for their formation. In this work, we performed an investigation devoted to the study of persistent radicals potentially involved in the formation of soot particles, by continuous wave and pulsed electron paramagnetic resonance. This work provides experimental evidence of the presence in nascent soot of highly branched, resonance-stabilized aromatic radicals bearing aliphatic groups, linked together by short carbon chains, and reinforced by non-covalent π-π interactions. These radicals appear to be highly specific of nascent soot and quickly disappear with the increasing soot maturity. Their presence in nascent soot could represent an underestimated health risk factor in addition to the already well documented effect of the high specific surface and the presence of harmful adsorbates.

## Abstract (web summary)

To combat the climate emergency, the development of less polluting technologies is crucial, but the mechanisms involved in the formation of soot particles are still not fully understood. Here, to fill this gap, the authors quantify and map persistent radicals on soot samples of different maturity extracted from flames using pulsed electron paramagnetic resonance.

## Introduction

The formation of carbonaceous nanoparticles (soot) in combustion is a major research topic due to their detrimental effect on human health and the environment. The World Health Organization estimates that soot particles released in the environment are responsible for about 4 million deaths per year worldwide^1^. After inhalation, ultrafine soot particles can enter the lungs and the circulatory system and be transported to the brain, liver, and heart where they can trigger respiratory, cardiovascular, and neurodegenerative effects^2^. In addition, polycyclic aromatic hydrocarbons (PAHs) adsorbed on soot particles and released in the body after inhalation are associated with mutagenic effects^3,4^. From an environmental point of view, soot particles are estimated to be the second most important contributor to climate change after CO_2_^5,6^. Once dispersed in the atmosphere, soot particles can affect the radiative balance by absorbing and scattering sunlight (direct effect)^7^. Moreover, after photochemical activation, they can act as condensation nuclei and trigger the formation of clouds and precipitations (indirect effect)^8^. In addition, soot particles trapped in ice decrease the albedo of snow and ice-covered surfaces and accelerate the melting of glaciers^9^.

The combustion of carbon-based fuels for transportation and power generation is currently the main source of soot particles in the atmosphere^10^. A large-scale ecological transition is considered a major technological, economic, and social challenge of the next 40 years^11^. Although this transition has already started in some industrial sectors, in particular for internal combustion engines, it will be much more complex for the maritime and aeronautical sectors for which the combustion of hydrocarbons will very likely remain the main source of power generation for many years to come.

To allow the development of less polluting technologies and limit emissions, it is essential to fully understand the mechanisms involved in the formation of soot particles. However, despite several decades of active research, a complete understanding of the physical–chemical processes governing the formation of soot particles has not been reached yet.

PAHs are well-known soot molecular precursors^12^. Soot formation through the stacking of moderate-size PAHs into clusters held together by van der Waals interactions and stabilized by C–C covalent bonds, or through the formation of cross-linked three-dimensional structures has been discussed for a long time^13^. Theoretical mechanisms have been more recently proposed that rely on the reversibility of the PAHs dimerization followed by the formation of chemical bonds between the dimers^14,15^, on self-sustaining reactions enabled by the thermodynamic stability of resonance-stabilized radicals^16^, or on the formation of rotationally activated dimers from the collision of an aromatic molecule and radical (*H-activation-C-addition* reaction mechanism)^17^. The presence of persistent radicals in nascent soot and their potential involvement in the soot inception process was pioneered by Cain et al.^18^ that concluded, based on experiments made in premixed flames using micro-FT-IR spectroscopy, that “[…] a higher temperature flame causes nascent soot to contain a larger number of these persistent free radical sites […]”. Regarding the role of aromatic radicals, Martin et al.^19^ studied the potential implications of σ and π aromatic radicals in the soot inception step and demonstrated using density functional methods that aromatic soot precursors containing localized π-radicals on rim-based pentagonal rings can form covalently stabilized π-stacked structures. The formation of bridged aromatics linked by aliphatic chains was additionally supported by the work of Adamson et al.^20^, reporting the formation of covalently bonded aromatic compounds (mostly pericondensed PAHs) formed in an atmospheric pressure inverse co-flow diffusion flame by high-resolution tandem mass spectrometry. Wang^13^ suggested that π-radicals could enhance bindings in PAH clusters. This suggestion is based on the assumption that the zigzag edges of PAHs possess localized π-electrons and thus exhibit radical or even biradical characteristics^21,22^. Therefore, PAHs would be able to form bonds via π-electron interactions with a strength similar to a covalent bond^19,23^. These structures would involve delocalized π-radicals (aromatic multicentre-linked hydrocarbons)^12^, localizable biradicals (aromatic zig-zag-linked hydrocarbons)^12^, and interactions between localized π-radicals (aromatic rim-linked hydrocarbons)^13^. Delocalized π electrons, capable of forming stabilized bonds in an aromatic molecule, confer significant thermodynamic stability to PAHs.

In parallel, Vitiello et al.^24^, based on experimental measurements in a sooting ethylene premixed flame by electron paramagnetic resonance (EPR), suggested that the clustering of aromatic structures is enhanced by the interaction of resonance-stabilized radicals, which form multicentric delocalized covalent bonds, intermediate between a pure covalent bond and a Van der Waals interaction. Localized π electrons can also promote clustering between aromatics through π-electron interactions on a stacked structure. Very recently, Commodo et al.^25,26^ reported direct evidence with high-resolution atomic force microscopy that nascent soot contains fused six-membered aromatic rings partially substituted with aliphatic chains. In addition, many of the observed structures contain five-membered rings on their periphery, while comparatively fewer five-membered rings are found embedded into the aromatic structure.

Despite the recent developments discussed above, experimental evidence on the structure and concentration of persistent radicals on soot remains scarce. To close this gap, we propose herein an experimental study based on EPR techniques to characterize the structure of the radicals found on soot at different reaction times during its formation and maturation in a flame environment. EPR gives acces to the concentration and some structural properties of the elusive persistent radicals present in the samples in trace amounts. Notably, the comparative analysis of the samples provides information on the early steps of particle formation from the molecular precursors.

Our investigation relies here on continuous wave electron paramagnetic resonance (CW-EPR) and for the first time to the best of our knowledge in a flame study on combining EPR imaging and pulsed EPR. In particular, this innovative approach provides valuable information for the characterization of the persistent radicals in nascent soot as well as some structural information about the aromatic units.

## Results and discussion

### Continuous wave EPR experiments

**Fig. 1:**
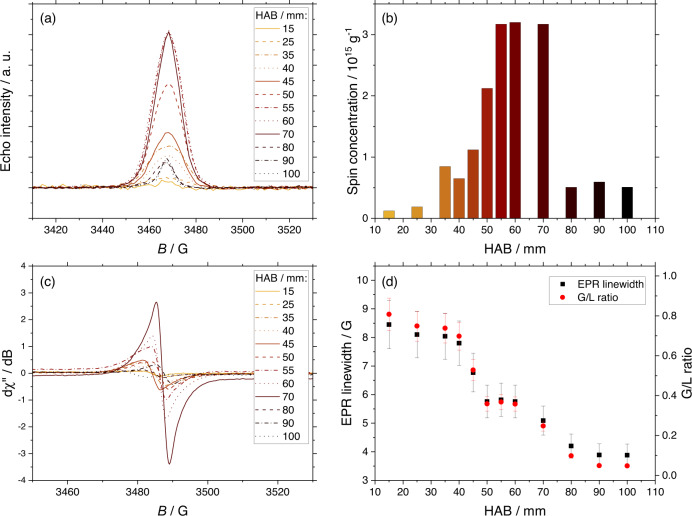
Continuous wave EPR experiments. **a** Echo detected spectra of the flame samples recorded at 5 K. **b** Spin concentration against sampling height above the burner (HAB). **c** CW-EPR spectra recorded at room temperature. **d** Evolution of the EPR linewidth and of the ratio of Gaussian/Lorentzian EPR spectral line shape. Error bars correspond to the maximum semi-deviation of the peak width measured at the peak FWHM.

Samples are extracted from a laboratory laminar diffusion methane flame at different heights above the burner (HAB) before and after the formation region of soot particles using a quartz microprobe, collected on silica paper and analyzed by CW-EPR (Supplementary Fig. 1). CW and echo detected field sweep spectra recorded for soot sampled against the HAB appear at *g* = 2.0034 that is indicative of the presence of carbon-centered radicals. An evolution of the EPR signal is observed as a function of the HAB. As shown in Fig. 1a and b, respectively, the echo intensity and the corresponding spin concentration continuously increase from 15 to 50 mm HAB, reach a maximum of around 3 × 10^15^ spin g^−1^ from 50 to 70 mm HAB, and then drastically decrease above 80 mm HAB. Previous experiments from our group showed that early soot particles, identified by in situ laser-induced incandescence using their graybody emission as a criterion to distinguish them from the gas phase, appear in 50–60 mm HAB^27–29^. Above this threshold, the soot volume fraction rapidly increases, reaches a maximum around 80 mm HAB, and then decreases when soot oxidation mechanisms become dominant.

The detailed analysis of the EPR data reported in Fig. 1c and d highlights the evolution of the linewidth against HAB. At low HAB, the EPR signals are significantly wider than at high HAB. In addition, the ratio between the Gaussian and Lorentzian contributions to the spectral line shape G/L also decreases at higher HABs. This evolution of the line width is associated with an evolution of the line shape from a Gaussian shape, corresponding to an inhomogeneous line and observed for all the HABs from 10 to 80 mm, to a Gaussian-Lorentzian line shape only observed at 90 and 100 mm HAB. The increase of the spin concentration concomitant with an increase of the Lorentzian contribution of the EPR line shape is consistent with the previous findings and analysis of Vitiello et al.^24^ in a sooting premixed flame of ethylene. In their paper, based on analogies with previous graphene and asphaltenes EPR studies, the authors interpreted this evolution as a possible rearrangement of the radical polyaromatic structures, containing first edge-localized nonbonding π-electrons^30^ and slightly evolving towards aromatic moieties with highly delocalized π-electrons^31,32^ or possibly due to stacking interactions between flat conjugated structures hosting unpaired electrons.

The line width in CW-EPR is influenced by spin clustering and is mainly affected by the unresolved hyperfine interaction between unpaired electrons with neighboring atoms^33^, and thus gives information on the nature of the analyte. A broad Gaussian line shape is typically associated with the presence and superposition of multiple paramagnetic species in particles^34–36^, whereas a narrow Gaussian line shape is related to the presence of strong electron–electron interactions^37^ and can result from the delocalization of unpaired electrons and/or the tendency for aromatic functional groups hosting radicals to stack up. Aumond et al.^38^ suggested that the evolution of the line width from narrow to wide indicates the evolution from a system of clustered spins to a system of homogeneous spins in connection with the formation of individual PAHs that grow and condense. Narrow line widths are generally characteristic of amorphous carbon-based materials^39^, or dehydrogenation during the maturation of the carbonaceous material^40^, and reveal changes in the spin systems of polyaromatic structures. This interpretation is in good agreement with the evolution of the line width beyond 70 mm HAB, which characterizes the transition from nascent to mature soot.

### EPR imaging experiments

**Fig. 2:**
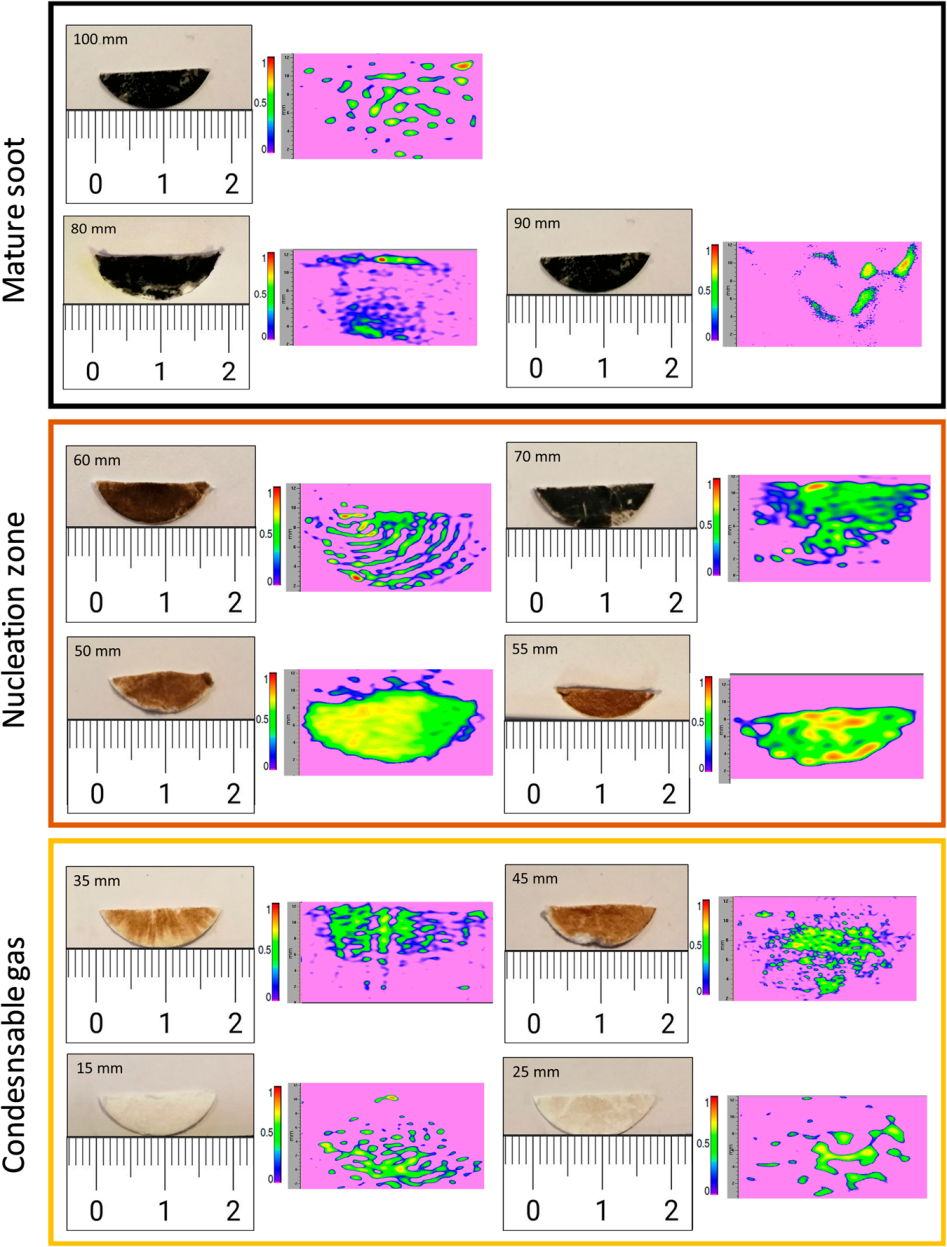
EPR imaging highlighting the distribution of persistent radicals in the collected samples. Pictures of the collected samples at different HAB and corresponding EPR images (512 × 512 image resolution, 0.05 mm pixel size). The colormap represents the spin concentration in relative unit, increasing from purple to red.

Figure 2 compares the EPR imaging with the pictures of the samples taken from the flame at the different HAB. The EPR images characterize the spatial distribution of the spin density of the carbon radicals.

Samples in 15–45 mm HAB are characterized by highly heterogeneous EPR images, with small and scattered signal islands that become larger and denser with the increasing HAB. We observe in the pictures of the samples that the increase of the EPR signal is associated with the appearance of a deposit of brownish matter. From 60–70 mm, the color of the deposited matter transitions from brown to black characteristic of soot particles, and corresponds to the appearance of incandescent soot particles in the flame^28^. Above 70 mm HAB, the signal intensity in EPR images begins to decline and small and scattered signal islands appear again. This observation suggests that persistent radicals would be contained in the brownish-colored material rather than in the more graphitized soot particles observed above.

### Pulsed EPR experiments

Pulsed EPR has been widely used in the last two decades to provide structural information on the radicals contained in primitive organic matter like the insoluble organic matter conserved in ancient sedimentary rocks and in carbonaceous meteorites^38,41–47^, but it has never been applied to flame soot. This powerful diagnostic is based on the hyperfine interaction of nuclei with spin ½ such as ^1^H, ^13^C, ^15^N, and ^31^P, or with spin 1 such as ^2^H and ^14^N. The analysis of the hyperfine interaction provides information on the nature of the chemical bonds, the structure of the electron ground state, and the electron–nucleus distance, and thus gives direct information on the structure of the radical. These data can be determined from the analysis of the two-dimensional HYSCORE spectrum (*HYperfine Sublevel CORrElation*) corresponding to the resulting two-dimensional set of echo modulations after Fourier transform.

In the case of alkylated polyaromatic radicals, ^1^H-HYSCORE spectra contain important chemical information on the nature of C–H bonds^41^. Three types of hydrogens can be distinguished in the molecular structure depending on their distance from the edge carbon atoms (Supplementary Fig. 2). First, aromatic hydrogens (directly bound to aromatic carbons, H_a_) are characterized by a significant dipolar interaction T in addition to the isotropic interaction *A*_iso_ resulting in peaks and ridges that are positively shifted from the anti-diagonal and exhibiting a typical “horn shape”. Second, benzylic hydrogens (bound to aliphatic carbons next to an aromatic system, H_b_) are characterized by a hyperfine interaction dominated by the isotropic coupling term *A*_iso_, with only a weak dipolar contribution T leading to pairs of spots along an axis *υ*_1_ = −*υ*_2_ crossing the diagonal *υ*_1_ = *υ*_2_, with a splitting equal to *A*_iso_. Finally, distant hydrogens (bound to aliphatic carbons next to other aliphatic carbons, H_d_) are characterized by a weak dipolar interaction giving a central peak along the diagonal *υ*_1_ = *υ*_2_ at the nuclear frequency of ^1^H *ν* = 14.5 MHz. These HYSCORE spectra might therefore advantageously be used to obtain information on the structure of polyaromatic radicals contained in nascent soot.

**Fig. 3:**
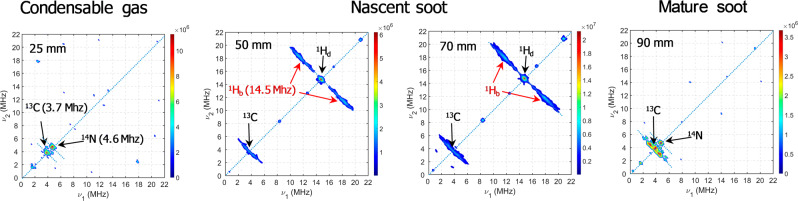
HYSCORE spectra against HAB and soot maturity. HYSCORE spectra of soot sampled at 25, 50, 70, and 90 mm in the central axis of the flame. Nascent soot is detected above 55–60 mm HAB.

The evolution of the HYSCORE spectra against HAB is reported in Fig. 3. To detect the hyperfine spectrum of both ^1^H and ^13^C in the same spectrum, all the spectra of Fig. 3 are recorded with the same interval between the two first π/2 pulses (136 ns)^48^. The HYSCORE spectra are characteristic of the hyperfine interactions providing information about the radical environment of condensable gas, nascent soot, and mature soot. The complete database of recorded spectra is available (Supplementary Fig. 3).

Figure 3 shows that the hyperfine interactions of ^1^H are all contained in the quadrant *υ*_1_ and *υ*_2_ > 0, which is characteristic of weak couplings between the unpaired electron and the neighboring nuclei. Second, the HYSCORE spectrum of the condensable gas shows the presence of ^13^C (3.7 MHz) and ^14^N (4.6 MHz), while no signal of ^1^H (expected at 14.5 MHz) is observed. The contribution of ^1^H only appears above 35 mm HAB and keeps increasing up to 70 mm HAB. In the mature soot above 80 mm HAB, the signal of ^1^H suddenly disappears, and a change in the shape of the carbon signal is representative of the signature of PAH macrocycles (graphitized soot). These results are consistent with the echo detected field sweep measurement results and highlight for ^1^H that the unpaired electron interacts with nuclear spins by two different hyperfine interactions. The intense peak in the *υ*_1_ = *υ*_2_ diagonal is characteristic of the hyperfine interaction with distant ^1^H and thus of aliphatic side chains, while the broad ridges on both sides of this central peak represent the hyperfine interaction with benzylic ^1^H. It is worth noticing that the typical spectrum of aromatic ^1^H is never observed for any sample. Aromatic ^1^H, for instance deriving from poorly branched organic radicals present in biogenic fossilized in cherts^41^, results in typical “horn-shaped” ridges. This strongly suggests that in the soot samples, many aromatic hydrogens have been substituted with aliphatic side chains, the sources of benzylic and distant hydrogens. By analogy with insoluble organic matter constituted of highly branched aromatic clusters linked by short and branched aliphatic chains^43,46,47^, the elongated shape of the signal ridge of benzylic hydrogens and the dominant signal of distant hydrogens in the HYSCORE spectra are indicative of branched aromatic radicals. This analysis, therefore, supports previous FTIR^18^ observations, showing a high number of aliphatic C–H bonds compared to that of aromatic C–H suggested to be mainly in the form of alkyl, alkenyl side chains or cross-linked aromatic units in the soot material^49^.

Also interesting to note are the drastic drop of the ^1^H signal and the change of shape of the ^13^C signal above 80 mm HAB. The evolution of the HYSCORE spectra highlights major differences in the nature of the radicals in nascent and mature soot and therefore appears to be a good indicator of the soot degree of maturation. In particular, the substituted aromatic radicals prominent in nascent soot are completely absent in mature soot. This information might be of crucial interest to address the health and environmental effects of soot particles. Indeed, there is a large body of research aiming to characterize the potential toxicity of persistent radicals associated with airborne particles from industrial and automotive emissions on human cells^50–52^. Nascent and mature soot are known to possess different physical–chemical properties, and thus different chemical reactivity^19^. In addition to their smaller size that results in higher total and specific surface and to the presence of PAHs adsorbates that can be released in the lungs after inhalation, the higher concentration of persistent radicals in nascent soot revealed by the analysis of the HYSCORE spectra might be at the origin of unexpected reactivity and thus to an underestimated risk factor to the health of these small particles in comparison with mature soot.

It is to be noted, about the presence of heteroatoms in the radicals, that the signal of ^15^N is only detected in the HYSCORE spectra in the condensable gas and mature soot, although mass spectrometry analyses often show the presence of heteroatoms in the structures of nascent soot^53^. Besides, pulsed EPR cannot provide any information on oxygen as ^16^O has a zero nuclear spin, and the low natural abundance of ^17^O combined with the already low atomic fraction of oxygen in soot consistently results in signals below the detection limit.

On the contrary, ^13^C gives an intense ridge in the HYSCORE spectra as also observed for carbonaceous meteorites and coals^41^. As shown by Ikoma et al.^44,45^, the ^13^C/^1^H intensity ratio increases with the number of equivalent carbon nuclei interacting with the unpaired electron as a larger number of carbons close to the aromatic system increases the probability for the electron spin to interact with a ^13^C nucleus. Therefore, the signal intensity of carbon has been shown to be sensitive to the molecular geometry by Ikoma et al.^44,45^ In particular, they established that the highest values of ^13^C/^1^H, close to 0.35 determined for specific coals, indicate a specific molecular association taking place around the radicals in the macromolecular structures of the coal. This association has been interpreted as a particular distribution of the unpaired electron between aromatic molecules connected by a noncovalent interaction like a π–π complex. Regarding our data, we determined a constant ^13^C/^1^H = 0.33 ± 0.03 in the region 40–70 mm HAB. This high value may therefore be indicative of the presence in nascent soot of persistent aromatic radicals substituted by short aliphatic chains and stabilized by π stacking interactions.

**Fig. 4:**
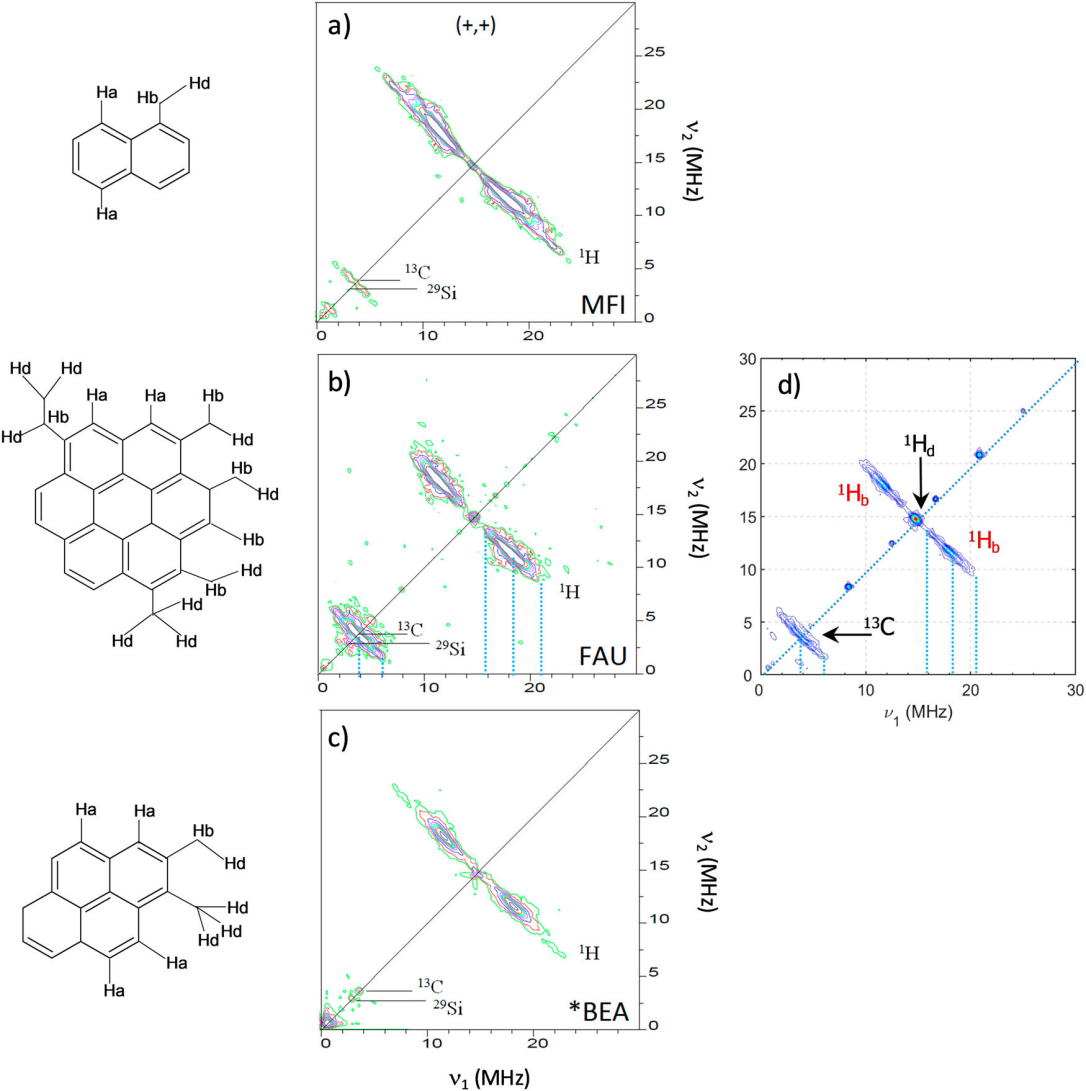
Estimation of the molecular structure of the radicals present in nascent soot. HYSCORE spectra of estimated internal radical compounds present in **a** FAU, **b** MFI, and **c** *BEA zeolites^48^ compared to the HYSCORE spectrum **d** of nascent soot obtained from our work. **a**–**c** are reprinted with permission from Ben Tayeb et al.^48^. Copyright 2023 Elsevier.

It is to be noted that HYSCORE spectra cannot provide information on the number of hydrogens of each type in the radicals. However, it is still possible to deduce the type of nucleus contributing to the signal. In addition, the ^13^C/^1^H ratio reflects size differences of aromatic fragments^41,48^. As recently shown by Ben Tayeb et al.^48^, the ^13^C/^1^H ratio and the shape of the HYSCORE spectra can also be used to reveal some specific features (size of the aromatic clusters and structural information) of the radicals present in various carbonaceous clusters. To this aim, they notably recorded the HYSCORE spectra of several cokes trapped in different-sized zeolites. According to their work, different aromatic radical clusters are characterized by distinctive HYSCORE spectra as shown in Fig. 4a–c. Moreover, it is observed that the ^13^C/^1^H ratio of the largest cluster (FAU) is notably higher than for the two lowest ones (MFI and BEA), due to the ^13^C signal being much more intense for the FAU cluster as can be seen in the HYSCORE spectrum in Fig. 4b in comparison with those reported in Fig. 4a and c. These differences are explained by the higher number of carbons located at a short distance from the polyaromatic unit that increases the probability for the unpaired electron to interact with a ^13^C nucleus. As a consequence, a higher ^13^C/^1^H ratio is characteristic of the number of equivalent carbons in a molecule, and indicative of high aliphatic branching from the aromatic units. For comparison, a typical HYSCORE spectrum obtained at 70 mm HAB shortly before the nascent soot disappears is shown in Fig. 4d.

The HYSCORE of nascent soot and of coke trapped in the FAU zeolite, respectively reported in Fig. 4b and d, share similar features. First, the ^1^H ridges (delimited by the blue dotted lines) characterizing benzylic hydrogens appear slightly shifted from the central signal at 14.7 MHz of distant hydrogens. Second, the shape of the ridge of the ^13^C signal is very similar to nascent soot. These similarities between the two HSYCORE spectra suggest that radicals in nascent soot are aromatic species roughly the size of coronene linked by short and branched aliphatic chains. This data is in good agreement with the previous work of Commodo et al.^54^, in which the authors conclude that the core of nascent soot particles is mainly constituted of polyaromatic units having a size of the order of 1 nm.

The same experiments described above were repeated with isotopically enriched methane (5% CD_4_). Nascent soot is characterized by the appearance of a coupling at the deuterium frequency (2.2 MHz). The ratio of the ^2^D/^1^H coupling is well within the expected gyromagnetic ratio of 6.6. This result confirms that the labeled sites correspond to formed radicals of the same nature. It is also important to note that in the presence of deuterium, mature soot still contains hydrogens which indicate a delayed reactivity by isotopic effect (Supplementary Fig. 4).

In conclusion, the data presented in this work provide experimental evidence of the presence in nascent soot of persistent aromatic radicals highly substituted by short aliphatic chains and stabilized by π stacking interactions. The structure of these radicals is consistent with resonance-stabilized π-radicals able to form multicenter π-bonds (“pancake bond”) already suggested to participate in the soot inception process by density functional theory calculations^19^. Our experimental data highlight that these resonance-stabilized radicals, which are absent from the condensable gas before soot formation, are formed and are essentially present in nascent soot to completely disappear from mature soot.

**Fig. 5:**
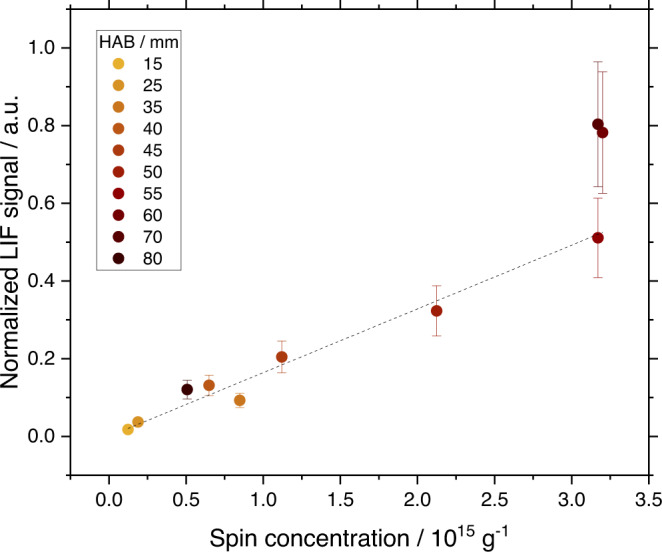
Correlation between visible fluorescence signal and spin concentration. Normalized laser-induced fluorescence signal (532 nm excitation)^28^ against spin concentration (data points) and linear fit (dashed line, *R*^2^ = 0.8943).

The implications of these findings are manifold. First, the optical properties of nascent and mature soot are known to differ^55^ and the radicals present in nascent soot might be at least partially responsible for such difference. As shown in Fig. 5, the spin concentration is found to be correlated to the concentration of soot precursors measured in situ in the flame by optical methods (laser-induced fluorescence^28^). Although the concentration of the individual fluorophores cannot be determined, this general correlation suggests that persistent radicals are related to the species fluorescing in the flame.

The absorbance function *E*(*m*) of nascent and mature soot is also well known to differ, with typical values varying from 0.2 for nascent soot and up to 0.4 for mature soot^55^. Despite being critical for the correct determination of the soot volume fraction during in situ measurements in combustion systems, the dependence of *E*(*m*) on soot maturity is still poorly understood. From our data, it might be speculated that the presence of radicals in nascent soot affects *E*(*m*) as well.

Furthermore, soot particles possess different physical–chemical properties that depend on their degree of maturation. Nascent soot is constituted of disordered and amorphous matter while mature soot is made of more graphitic-like matter^56^. In addition to the higher total and specific surface^57^ and H/C ratio^27^, the higher content of persistent radicals of nascent soot in comparison with mature soot might further contribute to the increased reactivity of nascent soot. According to our data, this reduced reactivity with the soot maturity might be linked to the decrease in the concentration of persistent aromatic radicals in the structure of soot. For all these reasons, nascent soot is expected to interact heavily with the environment and therefore to be more prone to cause deleterious health effects than mature soot, encouraging the implementation of adapted and more stringent strategies to regulate its emission.

## Methods

### Flame and sampling

Samples are collected from a 120 mm height laminar diffusion methane flame stabilized on a Gülder-type burner at atmospheric pressure^58^. This burner consists of a central injector supplied with 0.52 L min^−1^ of methan/e and surrounded by 86.6 L min^−1^ air flow (standard *p* and *T*). This flame is characterized by the stratified distribution of PAHs and soot through the centerline that allows sampling at different heights above the burner (HAB) to monitor the steps of soot formation. Soot and condensable gas are extracted from the flame using a quartz dilutive microprobe ending with a thin tip in which a 250 μm diameter orifice is obtained by abrasion and inserted radially into the flame. The small orifice allows for maintaining a constant pressure difference (≈500 mbar) between the flame and the probe during the sampling. Extracted species are diluted by nitrogen (0.5–5.0 L min^−1^) in the mixing chamber at the tip of the probe in order to reduce the collision probability in the sampling line and limit reactivity^27^. The collected species are deposited on glass microfiber filters using a 1/8” diameter impactor installed in a sample holder system downstream of the probe. An automatic pressure regulation system is located further downstream of the sample holder and consists of a HEPA filter, a Pfeiffer EVR116 regulation valve, and a pumping unit. The sampling time for the diffusion flame is set to 3 min per sample.

### Electron paramagnetic resonance (EPR)

Pulsed-EPR measurements were carried out at X-band (9 GHz) at 5 K with a Bruker Elexsys E580 spectrometer equipped with a helium flow cryostat. The concentration of organic radicals was derived from the CW and echo detected field sweep experiments (*π*/2–*τ*–*π*–*τ*) where the pulse lengths were set to 32 ns for the *π* pulse and 16 ns for the *π*/2 pulse with *τ* = 136 ns. *π*/2 and *π* represent the angles of rotation of the electron magnetization and *τ* the time delay between pulses.

Hyperfine interactions, i.e. interactions between the electron spin of the radical and neighboring nuclear spins, are measured using the two-dimensional four-pulse electron spin echo envelope modulation (ESEEM) (*π*/2–*τ*–*π*/2–*t*_1_–*π*–*t*_2_–*π*/2–*τ*). This technique is also referred to as hyperfine sublevel correlation spectroscopy (HYSCORE) whereby a *π* pulse is intercalated between two *π*/2 microwave pulses and the echo intensity is measured after the fourth pulse at fixed *τ*. The echo intensity exhibits modulations due to the presence of magnetic dipolar interactions between the unpaired electron of the radical and neighboring nuclear spins and is measured by varying *t*_1_ and *t*_2_ at fixed *τ*. This two-dimensional set of echo modulations gives, after Fourier transformation, a two-dimensional HYSCORE spectrum, which is represented by contour plots where the corresponding nuclear frequencies are obtained. This technique allows the detection of elements with non-zero nuclear spins such as ^1^H and ^13^C and the evaluation of the H/C ratio.

2D Spatial images were recorded using a continuous wave EPR X-band Bruker Elexsys E580 spectrometer operating at around 9 GHz and at room temperature. The microwave power was supplied into the resonator where the sample is placed and the modulation amplitude was set to 10 mW and 4 G, respectively. The signal was recorded with a field-of-view of 25 mm and a gradient strength of 175 G cm^−1^. The size of 2D YZ images was 512 × 512 pixels resulting in a pixel size of 0.05 mm. The image processing involves deconvoluting the acquired projections under a magnetic field gradient from the reference signal recorded without gradient. Both signals were back-projected using Fourier transformation, giving the spatial distribution of organic radicals in the samples.

## Supplementary information

Supplementary Information

## Data Availability

All relevant data are available from the authors upon request that can be addressed to Dr. Xavier Mercier.
