# Supplementary material for: Investigation of resonance-stabilized radicals associated with soot particle inception using advanced electron paramagnetic resonance techniques
Source: Commun Chem. 2023 May 24;6:99. doi: 10.1038/s42004-023-00896-4 (PMC10209094; doi:10.1038/s42004-023-00896-4)
Supplement: Supplementary file 1 — Supplementary Information [file 42004_2023_896_MOESM1_ESM.pdf]

Supplementary Information for

Investigation of Resonance-Stabilized Radicals Associated with Soot Particle Inception Using Advanced Electron Paramagnetic Resonance Techniques

Jessy Elias, Alessandro Faccinetto, Hervé Vezin*, Xavier Mercier*

*Correspondence to: Hervé Vezin (herve.vezin@univ-lille.fr) / Xavier Mercier (xavier.mercier@univ-lille.fr)

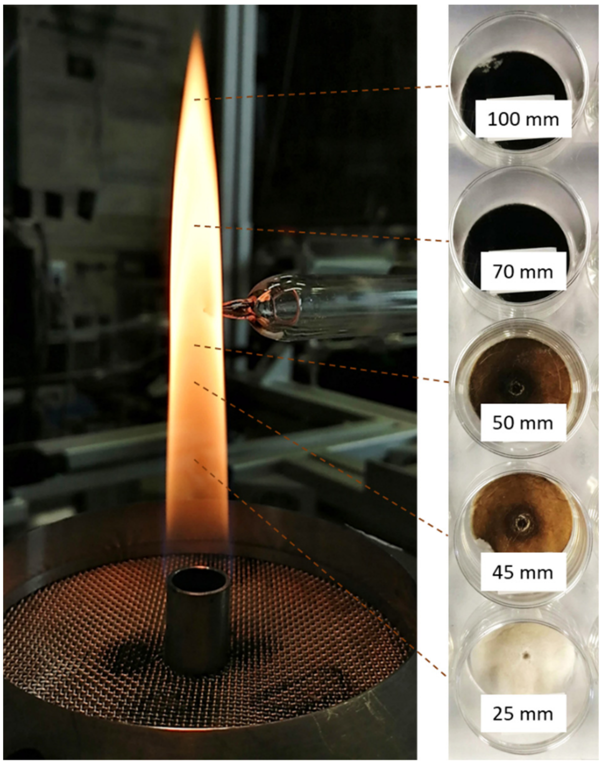

Supplementary Figure 1: Pictures of the investigated laminar diffusion methane flame showing the sampling probe and some of the collected samples on the side.

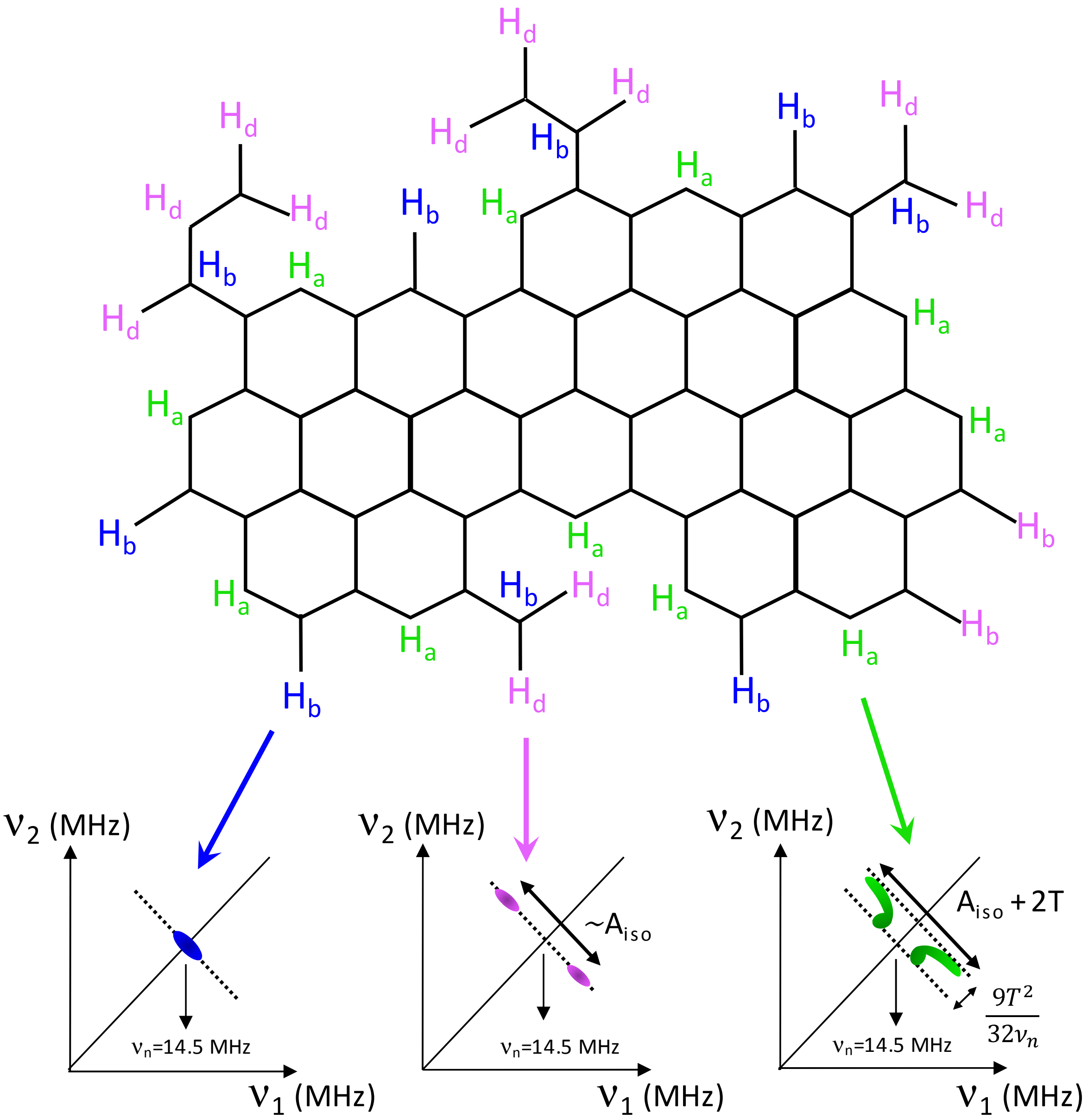

Supplementary Figure 2: Schematic representation adapted from Gourier et al.[1] of a hypothetical branched polyaromatic fragment in soot. The electron is delocalized on the $2p_{\pi}$ orbitals of aromatic rings. Distant hydrogen (H_d), benzylic hydrogen (H_b), and aromatic hydrogen (H_a) represent the three types of hydrogen atoms that can be identified in HYSCORE spectra. Schematic HYSCORE spectra for H_d, H_b, and H_a hydrogen atoms are represented with the indication of the hyperfine parameters. A_{iso} and T are the isotropic and dipolar components of the hyperfine interaction, respectively.

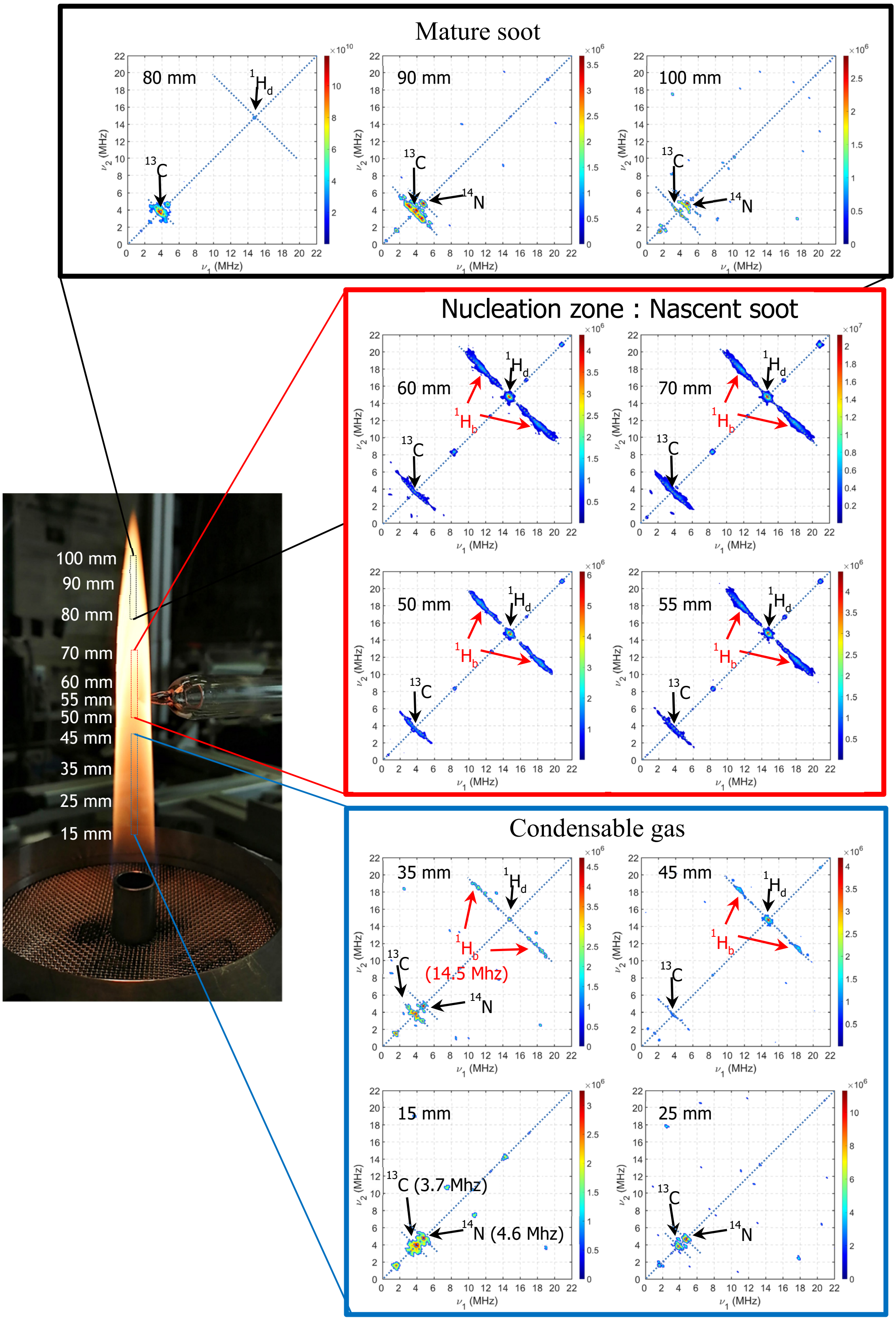

Supplementary Figure 3: Evolution of the HYSCORE spectra recorded at different HABs along the central vertical axis of the diffusion flame

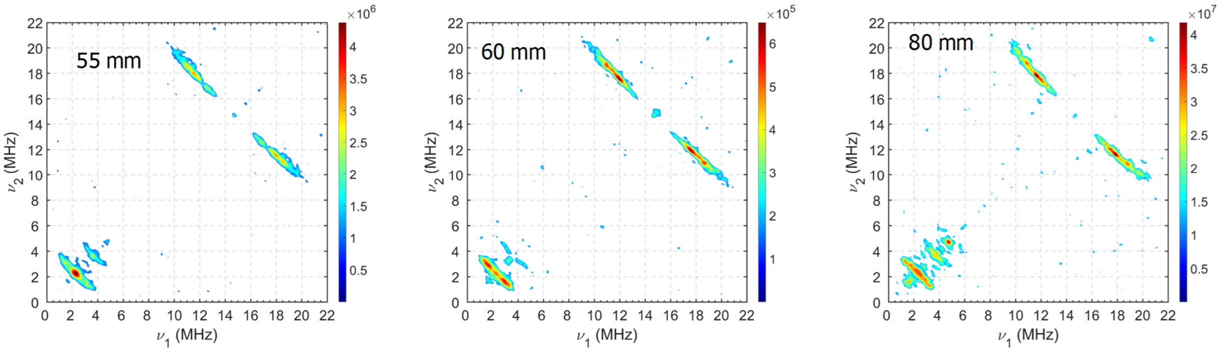

Supplementary Figure 4: Evolution of the HYSCORE spectra recorded for experiments performed with CD_4 at different HABs along the central vertical axis of the diffusion flame

Supplementary References

1. Gourier, D., Delpoux, O., Binet, L. & Vezin, H. Nuclear Magnetic Biosignatures in the Carbonaceous Matter of Ancient Cherts: Comparison with Carbonaceous Meteorites. Astrobiology **13**, 932–947 (2013).
